# Aspiration in Knee Joint Effusion after Traumatism

**Published:** 1902-02

**Authors:** R. Menger

**Affiliations:** San Antonio, Texas


THE
### TEXAS MEDICAL JOURNAL.

ESTABLISHED JULY, 1885.

PUBLISHED MONTHLY.—SUBSCRIPTION $1.00 A YEAR.

VOL. XVII. AUSTIN, FEBRUARY, 1902. No. 8.

### Original Contributions.

For Texas Medical Journal.

### Aspiration in Knee Joint Effusion After Traumatism.

BY R. MENGER, M. D., SAN ANTONIO, TEXAS.

I have no statistics on aspiration in cases of knee joint effusion from whatever cause, but, from the few cases I had (and undoubtedly others also), aspiration, done under proper care and with sterilized instruments, in large accumulations of fluid in this important joint, seems to be the proper as well as safe procedure. I will mention a case now under observation.

Some while ago a youngster, aged 15 years, was brought to my office in the following condition: His right knee joint was badly swollen in and around the popliteal region from traumatism with only slight swelling or bulging out of the joint around the patella. The region of the external condyle was so swollen and, on pressure, painful, that either a fracture or a rupture of a larger blood vessel suggested itself. As it was night time, I had the boy ordered home and applied lead water with laudanum, etc. Next morning the joint was still more swollen with more bulging out of the joint around the patellar region, and, of course, an effusion, perhaps hemorrhagic, suggested itself. The boy, I forgot to mention, in trying to step out of a wagon, slipped from the wheel he stepped upon and thereby was violently thrown against the iron tire, at the same time entangling his leg, which, his parents stated, was distorted when picked up. I put the leg in a temporary splint, kept up the lotion, and, seeing no improvement after the third day, but even more swelling of the knee joint, I concluded to aspirate and put the leg in an immobile apparatus. Accordingly, and with the kind assistance of Dr. R. L. Withers (who gave chloroform), I aspirated the knee joint at its most bulging point and drew off, by measure, five ounces of hemorrhagic effusion—nearly pure blood. During aspiration the joint sunk in and could be outlined as in its normal state. The joint was now wrapped in absorbent lint saturated with the lotion and put in a good splint supported by bandages, sandbags, etc. Before the aspiration, I may mention, his condition was quite alarming, with temperature of 102° and threatening knee joint inflammation, and, after the aspiration, it never went over 99°, and is still so at this time of writing (November 25, 1901).

Although a fracture could not be distinctly traced, there are strong indications of a deep-seated partial fracture around the external condyle of the femur with rupture of ligaments (and the aspirated intra-capsular hemorrhage) and of the knee joint. Except on pressure at the site of injury, he does not complain and the knee joint can be bent somewhat.

At the end of the third week the case was progressing favorably; no effusion in joint perceptible; less discoloration of skin at site of injury; the joint can now be manipulated freely and the boy is able to stand up and walk about the floor with but slight limping.

This case, as reported *per se* is of course nothing unusual, but I believe, considering the severity and usual consequences of joint injuries from intra-capsular blood effusion, and the usual after effects when treated without aspirating early, the matter is well worth while recording, and it is therefore, in this sense, submitted.

